# MicroRNA, Insulin Resistance, and Metabolic Disorders

**DOI:** 10.3390/ijms232416215

**Published:** 2022-12-19

**Authors:** Wan Lee

**Affiliations:** 1 Department of Biochemistry, Dongguk University College of Medicine, 123 Dongdae-ro, Gyeongju 38066, Republic of Korea; wanlee@dongguk.ac.kr; Tel.: +82-54-770-2409; 2 Channelopathy Research Center, Dongguk University College of Medicine, 32 Dongguk-ro, Ilsan Dong-gu, Goyang 10326, Gyeonggi-do, Republic of Korea

Insulin resistance is a significant health problem worldwide that contributes to a number of disorders, including type 2 diabetes and metabolic syndrome. Due to recent developments in molecular and cellular biology, microRNAs (miRNAs) have a become a popular topic of research, as they are now recognized as “key modulators” of physiological and pathological processes. Through the modulation of gene expression on the transcriptional, post-transcriptional, and translational levels, miRNAs can coordinate cell-to-cell communications and regulate a wide range of cellular functions. However, despite emerging evidence indicating that miRNAs play significant prognostic and therapeutic roles, the mechanisms through which they contribute to insulin resistance and metabolic disorders remain largely unknown.

This editorial provides a brief overview of the five papers published in the Special Issue of the *International Journal of Molecular Sciences*, entitled “MicroRNA, Insulin Resistance, and Metabolic Disorders.” This Special Issue aims to explore the novel functions and mechanisms of miRNAs that contribute to the etiologies of insulin resistance and metabolic diseases. In addition, it explores recent breakthroughs in miRNA research and the prospective applications of miRNAs as new biomarkers and therapeutic targets for metabolic disorders.

In the first article, “miR-143-null Is against Diet-Induced Obesity by Promoting BAT Thermogenesis and Inhibiting WAT Adipogenesis”, Jie Liu et al. reveal the crucial function of miR-143 in high-fat-diet (HFD)-induced obesity [1]. The authors found that the knockout of miR-143 (MiR-143KO) in mice alleviated HFD-induced obesity and improved insulin sensitivity by enhancing the thermogenesis of brown adipose tissue (BAT) and suppressing the adipogenesis of white adipose tissue (WAT). The miR-143 expression in HFD-induced obesity was found to be diminished in BAT but initially upregulated and then downregulated in WAT. In the state of HFD-induced obesity, the miR-143KO mice exhibited a substantial decrease in body weight, increased energy expenditure, suppressed insulin resistance, and enhanced glucose tolerance. Furthermore, miR-143KO decreased the adipose tissue weight, enhanced the mitochondrial quality and quantity, and stimulated thermogenesis and lipolysis in BAT but increased lipolysis and inhibited lipogenesis in WAT. Thus, this study contributes to our current understanding of the role of miR-143 in the adipose tissue and its significance for anti-obesity strategies in providing a new approach to obesity management by the suppression of miR-143 expression in BAT.

It has also become apparent that miRNAs play a crucial role in several cardiovascular diseases. In the second article, “Implication of miR-155-5p and miR-143-3p in the Vascular Insulin Resistance and Instability of Human and Experimental Atherosclerotic Plaque”, González-López et al. demonstrated the significances of miR-155-5p and miR-143-3p in the pathogenesis of atherosclerosis [2]. They found that miR-155-5p was increased while miR-143-3p was decreased in animal and human atherosclerotic lesions by evaluating dysregulated miRNAs in the aortas of apolipoprotein-E-deficient mice and human aortic and carotid tissues. In addition, they found miR-155-5p targets protein kinase B (AKT) and miR-143-3p targets the insulin-like growth factor type II receptor (IGF-IIR). Furthermore, they demonstrated that miR-143-3p overexpression decreased IGF-IIR and reduced apoptosis in vascular cells, whereas miR-155-5p overexpression decreased the AKT levels and its phosphorylation in vascular smooth muscle cells. As a result, the authors suggested that miR-155-5p and miR-143-3p participate in the development of insulin resistance and plaque instability by modulating the expressions of AKT and IGF-IIR and, thus, contribute to the etiology of atherosclerosis.

In the third article, “MiR-183-5p Induced by Saturated Fatty Acids Hinders Insulin Signaling by Downregulating IRS-1 in Hepatocytes”, Nguyen et al. revealed the critical function of saturated-fatty-acid (SFA)-inducible miR-183-5p in the regulation of the insulin signaling pathway and the pathogenesis of hepatic insulin resistance [3]. Based on the differential expression analysis of the miRNAs, they found a significant upregulation of miR-183-5p in palmitate-treated HepG2 cells and the livers of HFD-fed obese mice. A subsequent analysis showed that miR-183-5p directly targets the 3′UTR of insulin receptor substrate-1 (IRS-1) and epigenetically suppresses IRS-1 expression, thus inhibiting insulin signaling and insulin-induced glycogen synthesis in the hepatocytes. The results presented in this article provide new insights into the pathophysiology of obesity-associated hepatic insulin resistance.

Endothelial cell senescence is accelerated by hyperglycemia and contributes to diabetic complications. In the fourth article, “Hyperglycemia Promotes Endothelial Cell Senescence through AQR/PLAU Signaling Axis,” Wan et al. demonstrated the role of AQR in hyperglycemia-induced senescence and its underlying mechanism [4]. Using data from multiple aging model datasets, the authors discovered that high glucose and aging both upregulate AQR expression in a broad range of species and that AQR overexpression promotes endothelial cell senescence. AQR-overexpressing or -knocked down HUVECs were subjected to transcriptome analyses to determine the role of AQR in cellular senescence, and PLAU was selected due to its ability to enrich biological processes. The authors found that PLAU knockdown restored senescence-related phenotypes, endothelial cell activation, and inflammation in models induced by AQR or TNF-α. They demonstrated, for the first time, that the AQR/PLAU axis contributes to endothelial cell senescence, thus revealing a novel link between hyperglycemia and vascular dysfunction. This study may provide a means of preventing T2DM-induced premature vascular aging.

The diagnosis of diabetes mellitus at an early stage may prevent the progression of the disease and its complications. Hence, a considerable amount of research has been devoted to discovering and establishing the “signatures of miRNAs” that aid in the diagnosis and treatment of diabetes. In a review article entitled “miRNAs as Biomarkers in Diabetes: Moving towards Precision Medicine,” Angelescu et al. [5] summarize the current knowledge of the miRNA biomarkers of diabetes and miRNAs associated with direct cellular reprogramming strategies. In addition, the authors discuss the use of miRNAs for the cell therapy of damaged pancreatic tissues and as biomarkers of insulin resistance.
